# Comparative assessment of low-level laser therapy (LLLT) vs. topical application of amlexanox + lidocaine to treat recurrent aphthous ulcers (RAUs): A randomized controlled trial

**DOI:** 10.34172/joddd.2021.003

**Published:** 2021-02-13

**Authors:** Charu Mohan Marya, Jiksha Mehlawat, Ruchi Nagpal, Sakshi Kataria, Pratibha Taneja

**Affiliations:** Department of Public Health Dentistry, Sudha Rustagi College of Dental Sciences and Research, Faridabad, Haryana, India

**Keywords:** Amlexanox and lidocaine, Drug therapy, Low-level laser therapy, Randomized controlled trial, Recurrent aphthous ulcers

## Abstract

**Background.** The present study aimed to assess and compare the pain perception and ulcer sizes before and after applying low-level laser therapy (LLLT) and Amlexanox + lidocaine.

**Methods.** Twenty-six patients referring to the out-patient department of the institution and diagnosed with recurrent aphthous ulcers (RAU) were assigned to two groups to receive either LLLT or Amlexanox + lidocaine. In group 1, the patients were provided with amlexanox + lidocaine to apply topically four times daily. In group 2, the patients underwent LLLT with no tissue contact in inward circular motions for two cycles for 30 seconds. This study was registered in "the Clinical Trials Registry- India" (CTRI), with the registration number CTRI/2019/09/028222. The data were analyzed with SPSS 16.

**Results.** The intergroup comparison was performed using Mann-Whitney U test, and intragroup comparisons were made using Wilcoxon’s signed-rank test. The level of significance was set at *P* <0.05. The results showed that pain perception and ulcer size were significantly lower in group 2 subjects than group 1 subjects (*P* <0.05).

**Conclusion.** LLLT was more effective than amlexanox + lidocaine in the management of RAU. It is a cost-effective therapy for treating RAU.

## Introduction

India is the second largest country across the globe, with a population of >1.21 billion. Despite being the fastest-growing economy, it stands way behind in terms of education and standards of living and health.^1^ Oral health is a critical but overlooked element of overall health and well-being among adults.^2^ Despite being predominantly avoidable, oral diseases are common, with significant consequences on individuals.^3-5^ In the developing countries, many diseases have been on the increase with an ever-changing way of living or environmental circumstances, including diet and psychosocial and emotional stress. These effects contribute to the expansion and occurrence of various diseases, including arteriosclerosis, cancer, chronic liver disease/cirrhosis, asthma, obesity, depression, diabetes, hypertension, chronic obstructive pulmonary disease, heart diseases, stroke, recurrent aphthous stomatitis (RAS), and vascular dementia.^6,7^ In an otherwise healthy individual, few conditions develop in the oral mucosa. Ulceration, a break in the epithelium of oral tissues, often reveals nerve endings in the underlying lamina propria, leading to pain or soreness. Aphthous ulcerations, generally called “canker sores,” are the frequently occurring oral mucosal lesions that affect >20% of the population. The phrase “aphthous” is acquired from the Greek word “aphtha,” which means ulceration.^8^ Stanley described RAS under three distinct clinical versions, namely Miculiz’s aphthae, major RAS also referred to as periadenitis mucosa necrotica recurrens or Sutton’s disease, and herpetiform ulceration characterized by multiple ulcers which may be up to 100 in number.^9^

It is a marked oral condition of unspecified etiology characterized by more than two bouts of oral ulcers per year, not related to an underlying systematic abnormality.^10^

The recurrent aphthous ulceration (RAU) outbreak is associated with hereditary, psychological and socioeconomic stress, nutritional deficiencies, hormonal fluctuations, and immunologic deficiencies.^11-16^ There is some evidence that RAU is related to modified immunologic defenses or might be the symptoms of several pathogens rather than one. Due to the unknown etiology of such lesions, it is necessary to find a definitive cure.^17-19^ Several agents help manage aphthous ulcers, including antibiotics, anti-inflammatory and immune modulators, anesthetics, and alternative products.^20^ Topical agents, including over-the-counter preparations such as antiseptic mouthwashes, are usually recommended to most of the patients.^21^ Topical agents enhance reparative and regenerative processes, evoking the activation of aerobic metabolic processes and oxidative phosphorylation, enhancing in vitro oxygen consumption and accelerating the transport of glucose into the cells.

At present, the treatment modalities, such as topical antibacterial, anti-inflammatory, immunomodulatory or symptomatic modalities for the condition, are neither 100% reliable nor efficacious. Currently, there is no single well-established treatment for these common oral ulcers, and none of the existing treatments accelerate the healing process.^21-25^

Low-level laser therapy (LLLT) has been recently employed for the treatment of RAU due to its biomodulation properties, analgesic effects by stimulating the healing process, immediate pain relief without an overdose of medication or side-effects and prevention of recurrence.^8,26^ Apart from providing time and cost benefits to the patients, LLLT has also proved a safe and clinically effective therapy for treating RAU.^8^ This measure of treatment provides an opportunity to the dentists to widen the range of services provided in the practice and to ease the discomfort of patients rapidly and comfortably. In the era of evidence-based dentistry, a consistent procedure to evaluate the disease severity might prove beneficial in aiding the management of RAU. Therefore, this clinical trial aimed to compare LLLT and topical application of amlexanox + lidocaine in the management of RAU. We conducted a 12-week, parallel-design, two-armed randomized controlled trial to compare the efficacy of LLLT versus topical application of amlexanox + lidocaine in the management RAU.

## Methods

Subjects aged 20 years, diagnosed with recurrent minor aphthous ulcers, were recruited for treatment from August 2019 to October 2019. The subjects were assessed for eligibility following the inclusion and exclusion criteria. The inclusion criteria consisted of subjects having RAU in the oral cavity via the criteria proposed by Natah et al^27^ in 2004 and those who signed informed consent forms and followed the schedule. The exclusion criteria included subjects currently under therapy for RAU, on medications like analgesics and immunosuppressants, pregnant or lactating mothers/subjects, and with any other lesions in the oral cavity.

Sample size estimation was made using G*Power software (version 3.0). A minimum sample size of 26 (13 in each group) was sufficient for an alpha of 0.05, power of 80%, 0.6 as effect size. The subjects were randomly assigned to two groups [amlexanox + lidocaine (group 1)] and [LLLT (diode laser, wavelength: 976 nm, peak power: 5 W, frequency: 50/60 Hz (group 2)] using lottery method, with 13 subjects in each group. In group 1, the subjects were provided with amlexanox + lidocaine to apply topically four times each day and advised to dry the ulcers before ointment application. The patients were asked to moisten their finger, and a small amount of the paste (about ¼ inch) was squeezed on their wet fingertip; using gentle pressure, the paste was dabbed onto each ulcer. In group 2, the subjects underwent LLLT in a circular motion from outside to inside, consisting of two cycles with 100% duty for 30 seconds with a wavelength of 976 nm, starting the use at 320-µm fiber optic at a distance of 1‒3 mm, a peak power of 5 W, and a frequency of 50 Hz with no tissue contact.

### Post-treatment instructions

The patients were advised not to eat or drink for two hours and maintain routine oral hygiene practices of brushing twice a day with a fluoride toothpaste. Oral rinses, washes, use of NSAIDS, or vitamin supplements were not prescribed.

The visual analog scale (VAS) with a score range of 0‒10 was used to estimate the subjective pain associated with ulcers, and the ulcer size was estimated using a divider and a ruler with an accuracy of 1 mm. The data were collected at three time intervals: baseline, day 2 and day 3 in both groups. The results were entered in a study proforma prepared. The data were analyzed using SPSS 16. Graphs were prepared in Microsoft Excel. VAS scores were summarized as means and standard deviations. Owing to the ordinal nature of the study outcome variable, non-parametric tests of significance (for intergroup comparison, Mann-Whitney U test, and for intragroup comparison, Wilcoxon’s signed-rank test) were used for inferential statistics. The level of statistical significance was set at 0.05.

## Results

**Figure 1:**
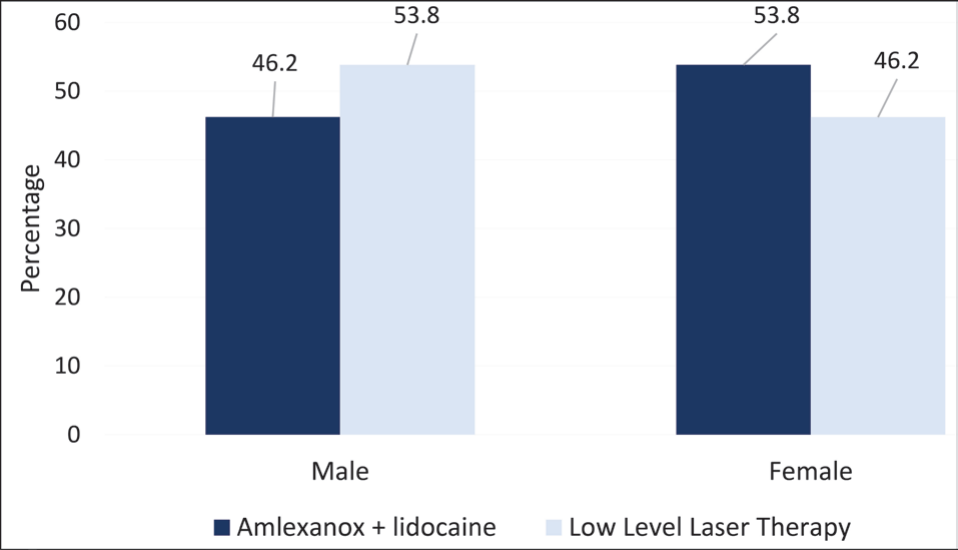
Genderwise distribution of subjects among amlexanox + lidocaine group and low level laser therapy group.

In the present study, the proportion of female patients was higher in the amlexanox + lidocaine group, whereas in the low-level laser group, male patients were more numerous (Figure 1).

**Figure 2:**
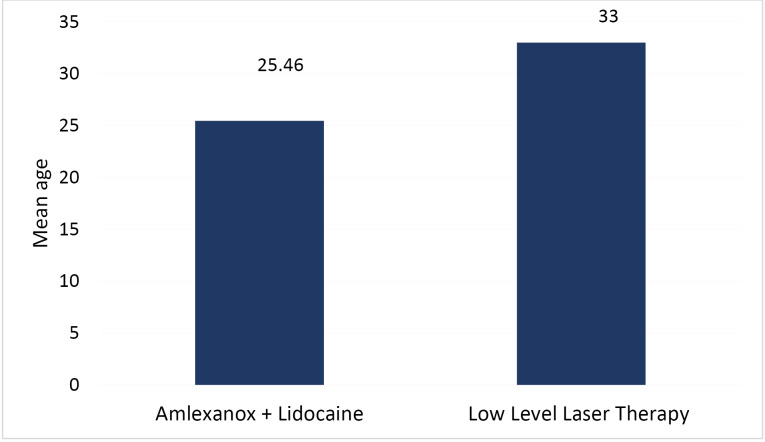
Mean age of subjects among amlexanox + lidocaine group and low level laser therapy group.

The mean age of subjects in the amlexanox + lidocaine group was 25.46 ± 5.41 years, with 33 ± 17.35 years in the LLLT group (Figure 2).

No significant age-wise or gender-wise differences were seen between ulcer areas and VAS scores for pain perception.

The means of ulcer areas of the subjects in group 1 on days 1, 2, and 3 were 18.92 ± 13.51, 16.52 ± 12.39, and 13.34 ± 11.17; in group 2, the means of ulcer areas on days 1, 2, and 3 were 18.80 ± 7.17, 10.46 ± 4.15, and 5.07 ± 3.36, respectively. The intragroup comparison of mean ulcer areas between the two groups showed a significant reduction from day 1 to day 3, whereas the intergroup comparison between the two different modalities showed a significant reduction on day 3 only (Table 1).

**Table 1:** Intragroup and inter-group comparison of mean ulcer area among amlexanox + lidocaine and low-level laser therapy groups

	**Group 1**	**Group 2**	**Inter-group:** ***P*** ^c^
Day 1	18.92	18.81	0.762, NS
Day 2	16.52	10.46	0.724, NS
Day 3	13.35	5.08	0.036*, SIG
Intra group: *P*^a^	0.001*, SIG	0.001*, SIG	
*P* ^b^: day 1 and day 2	0.248, NS	0.001*, SIG	
*P* ^b^: day 1 and day 3	0.013*, SIG	0.001*, SIG	
*P* ^b^: day 2 and day 3	0.010*, SIG	0.001*, SIG	

^a^ Friedman test, ^b^ Paired *t* test, ^c^ Mann-Whitney U test. Note: Level of significance set at *P* < 0.05. NS: Non-Significant, SIG: Significant.

The mean VAS scores for pain perception of subjects in group 1 on days 1, 2, and 3 were 8.23±1.58, 6.62±1.12, and 4.23±1.01, whereas in group 2, the mean VAS scores for pain perception on days 1, 2, and 3 were 8.92±1.89, 4.54±1.26, and 0.23±0.43, respectively. The intragroup comparison of mean VAS scores for pain perception in both groups showed a significant reduction from day 1 to day 3, whereas the intergroup comparison of the two groups showed a significant reduction on days 2 and 3 (Table 2).

**Table 2:** Intragroup and inter-group comparison of mean VAS scores for pain perception among amlexanox + lidocaine and low-level laser therapy groups

	**Group 1**	**Group 2**	**Inter-group:** ***P*** ^c^
Day 1	8.23	8.92	0.145, NS
Day 2	6.62	4.54	0.001*, SIG
Day 3	4.23	0.23	0.001*, SIG
Intra group: *P*^a^	0.001*, SIG	0.001*, SIG	
*P* ^b^: day 1 and day 2	0.51, NS	0.001*, SIG	
*P* ^b^: day 1 and day 3	0.012*, SIG	0.001*, SIG	
*P* ^b^: day 2 and day 3	0.045*, SIG	0.001*, SIG	

^a^ Friedman test, ^b^ Paired *t* test, ^c^ Mann-Whitney U test. Note: Level of significance set at *P* < 0.05. NS: Non-Significant, SIG: Significant.

**Figure 3:**
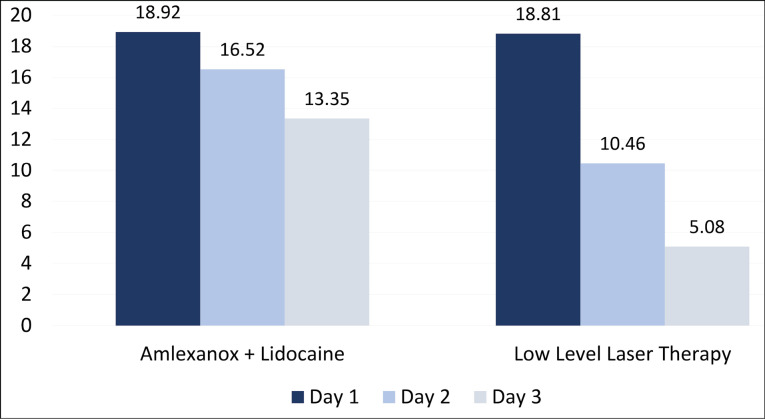
Intragroup comparison of mean ulcer area among amlexanox + lidocaine and low-level laser therapy groups.

**Figure 4:**
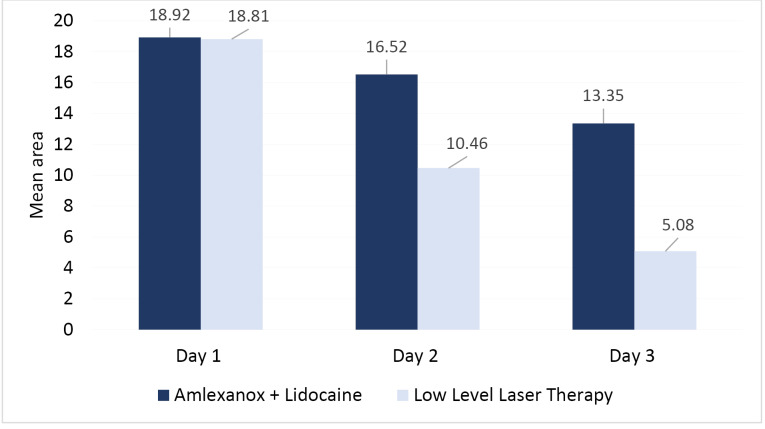
Intergroup comparison of mean ulcer area among amlexanox+ lidocaine and low-level laser therapy groups.

The mean percentage reduction in ulcer area from day 1 to day 2 (44.65%), from day 2 to day 3 (30.57%), and from day 1 to day 3 (75.21%) were more significant in the LLLT group than the amlexanox + lidocaine group (Figure 3). The mean percentage reductions of VAS scores for pain perception from day 1 to day 2 (48.46%), from day 2 to day 3 (49.23%), and from day 1 to day 3 (97.69%) were more significant in the LLLT group than the amlexanox + lidocaine group (Figure 4).

## Discussion

Numerous agents aid managing aphthous ulcers, including immune modulators, antibiotics, anesthetics, anti-inflammatory agents, and alternative products.^20^ Topical agents accelerate regenerative processes and oxidative phosphorylation, intensify oxygen consumption, and prompt the transport of glucose into the cells.^28^ Another mechanism proposed for pain relief is to modulate pain perception by modifying nerve conduction through the release of endorphins and enkephalins.^29^

The main concern of treatment is to reduce pain and ulcer sizes.^8^ The results of the present study showed that the magnitude of reducing the pain intensity and size of aphthous ulcers was higher with LLLT compared to amlexanox + lidocaine.

Healing is the main characteristic of LLLT, including three principal factors. Firstly, the laser increases adenosine triphosphate (ATP) production, leading to the augmentation of mitotic activity and increased protein synthesis by mitochondria, resulting in greater tissue regeneration in the repair process. Secondly, cell multiplication is facilitated by the stimulation of microcirculation, leading to an increase in the delivery of nutritional elements correlated with the increased speed of mitosis. Lastly, neoangiogenesis occurs from the pre-existing vessels.^8^

In the present study, a significant reduction in ulcer area and pain was detected with LLLT compared to amlexanox + lidocaine. Similar results were reported by De Souza et al^30^ in 2010, revealing a reduction in pain intensity in the same appointment after laser treatment, and complete regression of the lesion occurred after four days in 75% of the patients. Khademi et al^31^ reported similar benefits of rapid healing and reduction in pain after LLLT of RAS. In 2014, Darshan et al^32^ showed that 5% amlexanox could reduce the frequency, duration, and symptoms associated with the aphthous ulcers with no side effects attributed to the drug. Similarly, LLLT was efficacious in alleviating pain and decreasing the healing time in the course of treatment of aphthous ulcers in a study by Aggarwal et al.^33^ In contrast to these studies, a study by Jijin et al^34^ on LLLT and 5% amlexanox showed that both had equal efficacy in decreasing pain and ulcer size associated with minor aphthous outbreaks.

There were no side effects of LLLT and amlexanox + lidocaine in the present study. As there are no medications, the side effects and likelihood of overdose or adverse drug effects could also be avoided. Hence, it can be concluded that LLLT is a cautious and clinically successful therapy for RAU. The outcomes of the present study that standout are immediate and lasting pain relief and accelerated ulcer healing. The limitations of the study include a subjective evaluation of pain perception. Although healing occurs through medications and laser therapy, it mainly depends on host immune response and microbial interactions.

## Conclusion

It is concluded that LLLT is more effective than amlexanox + lidocaine in the management of RAU. It is a safe and clinically effective therapy for the treatment of RAU.

## Authors’ Contributions

MCM conceived the idea and contributed to manuscript reviewing. MJ was responsible for literature search, experimental studies, and manuscript preparation. NR was responsible for the design and defining the intellectual content. KS was responsible for clinical studies and data acquisition. TP was responsible for data analysis and manuscript editing.

## Acknowledgments

The authors would like to thank the patients for their consistent support and contribution.

## Funding

Self-funded.

## Competing Interests

The authors declare no competing interests with regards to the authorship and/or publication of this article.

## Ethics Approval

Ethical clearance was sought from the Institutional Ethical Committee (IEC) of the institute after presenting the aims and procedures of the study. Informed consent was taken from all the subjects. This study was registered in “The Clinical Trials Registry- India” (CTRI) with registration number CTRI/2019/09/028222.
